# Preoperative Embolization Reduces the Risk of Cathecolamines Release at the Time of Surgical Excision of Large Pelvic Extra-Adrenal Sympathetic Paraganglioma

**DOI:** 10.1155/2012/481328

**Published:** 2012-09-04

**Authors:** Nicola Di Daniele, Maria Paola Canale, Manfredi Tesauro, Valentina Rovella, Roberto Gandini, Orazio Schillaci, Federica Cadeddu, Giovanni Milito

**Affiliations:** ^1^Department of Internal Medicine, Rome Tor Vergata University, Viale Oxford 81, 00133 Rome, Italy; ^2^Department of Radiology, Rome Tor Vergata University, Viale Oxford 81, 00133 Rome, Italy; ^3^Department of Nuclear Medicine, Rome Tor Vergata University, Viale Oxford 81, 00133 Rome, Italy; ^4^Department of Surgery, Rome Tor Vergata University, Viale Oxford 81, 00133 Rome, Italy

## Abstract

A 30-year-old woman with severe hypertension was admitted to the hospital with a history of headache, palpitations, and diaphoresis following sexual intercourse. Twenty-four hour urinary excretion of free catecholamines and metabolites was markedly increased as was serum chromogranin A. Computed tomography scan revealed a large mass in the left adnex site and magnetic resonance imaging confirmed the computer tomography finding, suggesting the presence of extra-adrenal sympathetic paraganglioma. I-metaiodobenzyl guanidine scintigram revealed an increased uptake in the same area. Transcatheter arterial embolization of the mass resulted in marked decreases in blood pressure and urinary excretion of free catecholamines and metabolites. Surgical excision of the mass was then accomplished without complication. Preoperative embolization is a useful and safe procedure which may reduce the risk of catecholamines release at the time of surgical excision in large pelvic extra-adrenal sympathetic paraganglioma.

## 1. Introduction 

Previous reports have described the use of trancatheter arterial embolisation (TAE) in patients with pheochromocytoma. Preoperative embolization in both adrenal and extra-adrenal locations (including thoracic, intrapericardial, and left atrium [1–3]) has been shown to reduce the risk of catecholamines release during manipulation of the tumor at the time of surgery. Management of massive retroperitoneal haemorrhage has also been successfully performed with this procedure [4, 5]. Treatment of malignant pheochromocytoma [6] or its metastatic lesions [7–12], particularly liver metastasis [13, 14], has been performed too. Percutaneous transcatheter interventional procedures are also increasingly being employed in the management of gynaecological and obstetric disorders [15–17], including fibroids and postpartum haemorrhage [18, 19], and the management of tumor-related bleeding caused by both gynaecological malignancies and tumor metastatic to the vulvovaginal area [20, 21].

No previous reports have described of the use of TAE in the management of pelvic extra-adrenal sympathetic paraganglioma. The aim of the present study is to describe the usefulness of TAE in the preoperative management of pelvic extra-adrenal sympathetic paraganglioma in a young female patient.

## 2. Case Report

A 30-year-old caucasian woman was admitted to the hospital following syncope associated with a pounding headache, epistaxis, and blurred vision. 

Her past medical history included uncomplicated removal of an ovarian cyst and cholecystectomy at age 16 and 22 years, respectively. She delivered vaginally two children at age 23 and 25 years. She was in good health until a year prior to this presentation when she developed frequent postprandial vomiting and weight loss. Type 2 diabetes mellitus was diagnosed by oral glucose tolerance test and she was treated with metformin (1500 mg od). The patient also reported frequent attacks of severe headache, particularly during and following sexual intercourse. More recently, she had developed nocturia.

Physical examination revealed severe hypertension (240/150 mmHg), tachycardia (150 bpm), and diaphoresis. Physical examination was otherwise unremarkable. Blood chemistry demonstrated hyperglicemia (16.22 mmol/L), elevated glycosylated haemoglobin (57 mmol/mol, normal range: 20–40 mmol/mol), normal serum electrolytes, and liver and renal function. The EKG only showed sinus tachycardia (150 beats per minute). 

The patient underwent biochemical screening for cause of secondary hypertension. Due to the severity of hypertension, a washout period from antihypertensive medications was not obtained. Serum chromogranin A (CgA) was elevated at 3819 *μ*g/L (normal range: 19–98), and 24 hour urinary excretion of both free catecholamines (CATH) and metabolites was markedly increased. Urinary epinephrine (E) was 1876 nmol/d (normal limit 3–109 nmol/d); norepinephrine (NE) was 130883 nmol/d (normal range 136–621 nmol/d); normetanephrine (NMETH) was 196560 nmol/d (upper normal limit 2129.4 nmol/d); metanephrine (METH) was 2621 nmol/d (normal range 0–1622 nmol/d); vanilmandelic acid (VMA) was 716 *μ*mol/d (normal range 9.09–33.9 *μ*mol/d). Twenty-four hour urinary cortisol was slightly elevated: 712 nmol/d (normal range: 77.2–590.7 nmol/d). Plasma renin activity (1.19 *μ*g/L/h), plasma aldosterone (6.81 nmol/L), and plasma cortisol (527.5 nmol/L) were within normal limits. There was no left ventricular hypertrophy on transthoracic echocardiography. 

**Figure 1:**
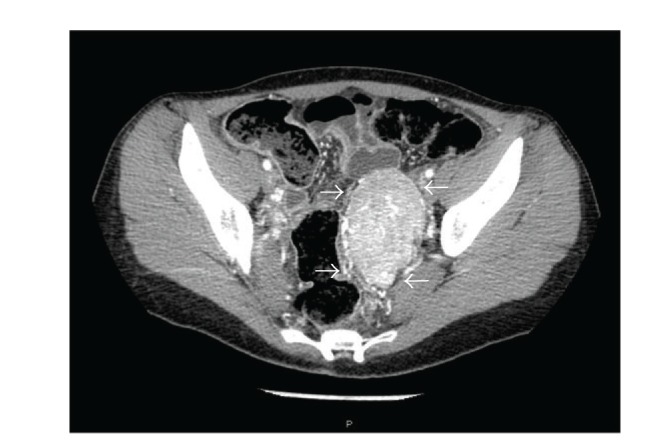
Enhanced pelvic CT scan showing a large mass in the left adnex site.

**Figure 2:**
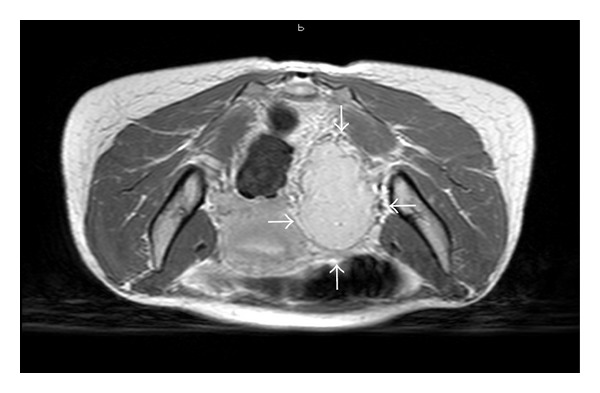
Pelvic MRI suggestive of a parauterine left site extra-adrenal sympathetic paraganglioma in the images obtained after i.v. injection of gadolinium: axial view.

**Figure 3:**
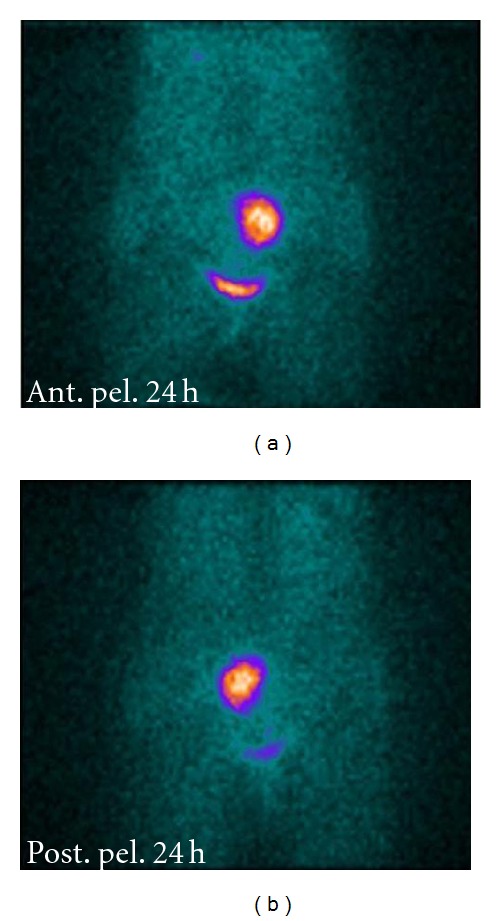
I-MIBG scintigraphy showing accumulation of radiopharmaceutical in the left paramedian site of the pelvis.

A computed tomography (CT) scan revealed no adrenal abnormalities, but a large mass in the left adnex site (7 cm of diameter) (Figure 1) was detected. Magnetic resonance imaging (MRI) was suggestive of an extra-adrenal sympathetic paraganglioma (Figure 2). Whole-body I-metaiodobenzyl guanidine (MIBG) scintigraphy detected increased uptake in the same area (Figure 3) without any other pathological activity elsewhere. Biochemical screening for multiple endocrine neoplasia was negative. Antihypertensive drugs were used to manage hypertension, to control associated cardiovascular symptoms, and to prepare the patient for surgery. The patient was initially started on labetalol i.v. (100 mg in 250 mL saline at 30 mL/h), increasing doses of oral doxazosin were then added (up to 8 mg/day) while labetalol was tapered. Subsequently, oral atenolol (100 mg/day) was added to the therapy. 

**Figure 4:**
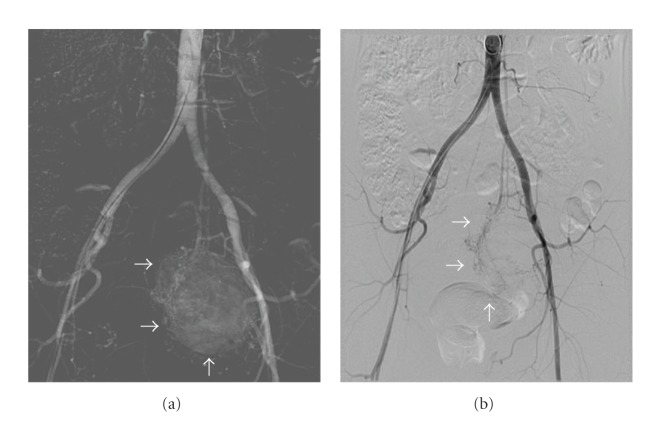
Angiography before and after TAE: tumour traces decreased in the target lesion after TAE.

**Figure 5:**
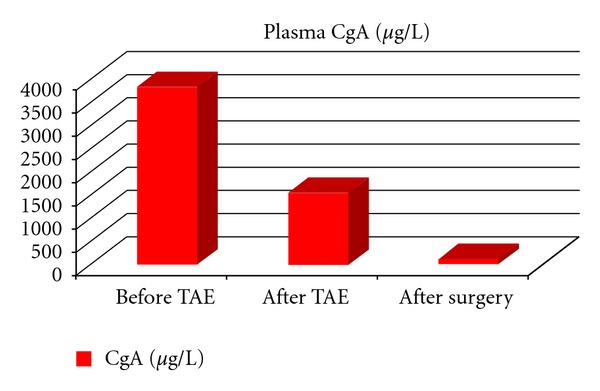
Changes in plasma CgA following TAE and surgery.

**Figure 6:**
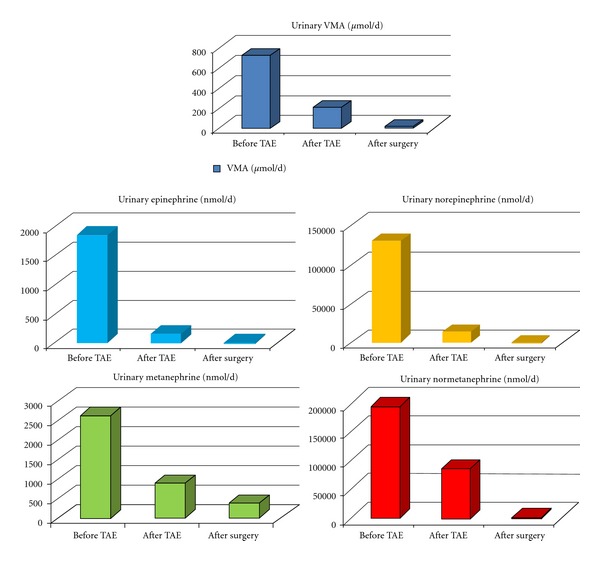
Changes in 24 h urinary VMA, free CATH (E+NE), and Metanephrines (METH+NMETH) following TAE and surgery.

**Figure 7:**
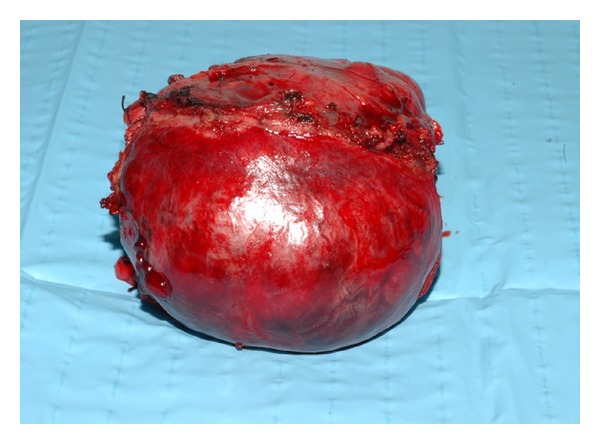
Resected paraadnexal extra-adrenal sympathetic paraganglioma.

Percutaneous transarterial embolization (TAE) of the mass was successfully performed via the inferior mesenteric artery (Figure 4). This resulted in a marked decrease in plasma CgA (1531 *μ*g/L) (Figure 5) and 24-hour urine excretion of VMA (202 *μ*mol/d) and total free CATH and Metanephrines (102074 nmol/d) (Figure 6). Laparotomic excision of a para-adnexal mass was performed after dissection of the tumour from the left iliac vessels (Figure 7).

Plasma CgA and 24-hour urine excretion of VMA and total free CATH and Metanephrines values following surgery are also shown in Figures 5 and 6, respectively.

Histological findings were consistent with pheochromocytoma (paragangliomas synaptophysin, positive; CroA, positive; S-100, very few positive cells; Ki67, approximately 3% of positive cells). In the postoperative period, the patient did not require treatment for hypotension or hypoglycaemia. At followup, she is normotensive and has normal glucose tolerance on no antihypertensive or hypoglycaemic medications.

## 3. Discussion 

In this case, despite the presence of the triad of headache, tachycardia and diaphoresis, which has been shown to have very high sensitivity for the diagnosis of pheochromocytoma [22], the temporal association of these features with sexual intercourse probably caused the delay of the diagnosis. This manifestation reflected its extra-adrenal location. Besides, this large extra-adrenal phaeochromocytoma was not associated with familial syndromes and was not metastatic. This variety of clinical features can explain why the differential diagnosis is often demanding [22]. In this patient, the diabetes initially predominated the clinical picture and disappeared after the removal of the tumour.

Although the onset of the tumor was unknown, its large size is consistent with its presence for at least few years. Interestingly, in this patient neither prior surgery, nor at the induction of anaesthesia nor during vaginal deliveries, hypertensive crises happened.

Hypertension was initially controlled with labetalol iv, an alpha-beta blocker agent, and then oral doxazosin, a selective alpha-receptor antagonis, added for few days prior to surgery. Many studies suggest that a long-lasting alphablockade is no longer necessary to prepare patients for surgery. Newell et al. reported that preoperative adrenergic blockade did not prevent severe intraoperative hypertension and that prolonged periods of preparations were not more effective in preventing intraoperative tachycardia and ventricular arrhythmias [23]. Boutros et al. reported similar perioperative results whether or not patients received preoperative alpha blockade [24]. Moreover, in a study of 114 patients who underwent removal of pheochromocytoma, fewer perioperative complications were observed in those not given alpha-blockers [25]. These results reflect the advances in anaesthetic and monitoring techniques and the availability of fast-acting drugs capable of correcting sudden changes in cardiovascular hemodynamics [22].

## 4. Conclusion

To our knowledge, this is the first report of the use of TAE in the management of pelvic extra-adrenal sympathetic paraganglioma. Marked decrease in circulating levels of catecholamines following TAE may be associated with lower risks of haemodynamic fluctuations during surgical manipulation of the tumour and in the postoperative period. 

This paper demonstrates that TAE may have an important role in the preoperative management of large pelvic extra-adrenal sympathetic paragangliomas.
